# ASO Author Reflections: Opportunity to Improve Outcomes in Patients with Gastric Cancer

**DOI:** 10.1245/s10434-025-18689-5

**Published:** 2025-11-11

**Authors:** Arsha Ostowari, Maheswari Senthil

**Affiliations:** 1 Department of Surgery, University of California, Irvine Medical Center, Orange, CA USA; 2 Division of Surgical Oncology, Department of Surgery, University of California, Irvine Medical Center, Orange, CA USA

Gastric cancer peritoneal metastasis is associated with extremely poor survival.^1^ Unfortunately, radiographically occult peritoneal metastasis is present in 30-40% of newly diagnosed gastric cancer cases.^2–4^ Therefore, staging laparoscopy (SL) with peritoneal cytology is considered a critical component of the staging workup and the National Comprehensive Cancer Network strongly recommends SL with cytology for adequate staging of gastric cancer.^5^

Regrettably, we found that the compliance with SL prior to neoadjuvant systemic therapy (NST) in patients that were referred/treated at our tertiary academic institutions was only 30%.^6^ The poor compliance with SL is even more worrisome as the incidence of peritoneal metastasis in patients who underwent SL was as high as 38%. Not surprisingly, in the patients in whom SL was omitted prior to NST, the median time to recurrence after gastrectomy was 11 months and the most common site of recurrence was the peritoneum. These results raise serious concerns about the management approach of patients with newly diagnosed gastric cancer particularly in the context of increasing incidence of gastric cancer in the United States. Surveillance, epidemiology and end results (SEER) data show that since 2019 the annual percentage incidence of new gastric cancer cases has been increasing by 3.6% in both sexes combined, and by 6.2% in females alone. Even more alarming is that the highest percentage change in increasing incidence (4.3%) is seen in people < 50 years of age.^7^ These trends are worrisome and further amplify the issue about inadequate staging and treatment in the youngest group of patients in whom there is a tremendous impact with missed opportunity for meaningful extension of life.

While the reasons for poor compliance with SL are difficult to determine, it is likely multifactorial and possibly influenced by institutional and provider practice preferences, insurance barriers, access issues, and the sense of urgency to start systemic therapy. Nevertheless, the importance of identification of metastatic disease and formulating a treatment plan that is stage appropriate cannot be overemphasized. In the past decade, treatment of gastric cancer, particularly metastatic gastric cancer has evolved with the addition of immune checkpoint inhibitors and targeted therapies, and these combinatorial approaches are associated with improved overall survival.^8,9^ Furthermore, the utilization of regional therapies in combination with systemic therapy through clinical trials are also options for these patients. For example, in the United States, EA2234 STOPGAP II (NCT 07001748) is a randomized controlled clinical trial evaluating the role of intraperitoneal paclitaxel combined with systemic therapy in patients with synchronous gastric cancer peritoneal metastasis (CYT+ or peritoneal carcinomatosis).^10^ Hence, regardless of the reasons behind the poor compliance, under staging patients would deprive them from receiving the appropriate first line systemic therapy or be considered for clinical trials.

Significant efforts are urgently needed to improve compliance with SL with cytology. These efforts should include education through community outreach, improving multidisciplinary care with the involvement of surgeons at the onset of cancer care, and eliminating barriers to care. An additional strategy is to develop an artificial intelligence (AI) deep learning (DL) model based on staging computed tomography scans and clinical risk factors to diagnose peritoneal disease. Such a model can be applied at the time of diagnosis or even after the initiation of NST to obtain an objective risk assessment regarding the of presence of peritoneal metastasis even when a SL with cytology was not performed at the time of diagnosis. Our team has developed an AI/DL model for peritoneal metastasis which is currently being validated in a large international cohort of patients with a plan for clinical translation in the near future.

Although there is significant work ahead of us to improve the outcomes of patients with gastric cancer peritoneal metastasis, the first step is to become aware of the current state of poor compliance with SL with cytology and failure to detect peritoneal metastasis. Undoubtedly, with the advances in management of patients with metastatic gastric cancer, increasing compliance with SL will lead to better management and improved patient outcomes.

## References

[1] Thomassen I, van Gestel YR, van Ramshorst B, et al. Peritoneal carcinomatosis of gastric origin: a population-based study on incidence, survival and risk factors. *Int J Cancer*. 2014;134(3):622–8. 10.1002/ijc.28373. 23832847 10.1002/ijc.28373

[2] Bentrem D, Wilton A, Mazumdar M, Brennan M, Coit D. The value of peritoneal cytology as a preoperative predictor in patients with gastric carcinoma undergoing a curative resection. *Ann Surg Oncol*. 2005;12(5):347–53. 10.1245/ASO.2005.03.065. 15915368 10.1245/ASO.2005.03.065

[3] Dobrzhanskyi O, Gaskill CE, Kizub D, et al. Utilization of staging laparoscopy for patients with gastric cancer in ukraine. *J Surg Oncol*. 2025;132(1):65–9. 10.1002/jso.28095. 39828960 10.1002/jso.28095

[4] Ikoma N, Blum M, Chiang YJ, et al. Yield of staging laparoscopy and lavage cytology for radiologically occult peritoneal carcinomatosis of gastric cancer. *Ann Surg Oncol*. 2016;23(13):4332–7. 10.1245/s10434-016-5409-7. 27384751 10.1245/s10434-016-5409-7

[5] Ajani JA, D’Amico TA, Bentrem DJ, et al. Gastric cancer, version 2.2022, NCCN clinical practice guidelines in oncology. *J Natl Compr Canc Netw*. 2022;20(2):167–92. 10.6004/jnccn.2022.0008. 35130500 10.6004/jnccn.2022.0008

[6] Ostowari A, Chen KT, Hasjim BJ, et al. Underutilization of Staging Laparoscopy Prior to Neoadjuvant Systemic Therapy in Gastric Cancer. *Ann Surg Oncol*. 2025. 10.1245/s10434-025-18547-4. 41107646 10.1245/s10434-025-18547-4 PMC12765731

[7] Cancer of the Stomach - Cancer Stat Facts. SEER. Accessed October 18, 2025. https://seer.cancer.gov/statfacts/html/stomach.html

[8] Janjigian YY, Shitara K, Moehler M, et al. First-line nivolumab plus chemotherapy versus chemotherapy alone for advanced gastric, gastro-oesophageal junction, and oesophageal adenocarcinoma (CheckMate 649): a randomised, open-label, phase 3 trial. *The Lancet*. 2021;398(10294):27–40. 10.1016/S0140-6736(21)00797-2. 10.1016/S0140-6736(21)00797-2 PMC8436782 34102137

[9] Shah MA, Shitara K, Ajani JA, et al. Zolbetuximab plus CAPOX in CLDN182-positive gastric or gastroesophageal junction adenocarcinoma: the randomized, phase 3 GLOW trial. *Nat Med*. 2023;29(8):2133–41. 10.1038/s41591-023-02465-7. 37524953 10.1038/s41591-023-02465-7 PMC10427418

[10] ECOG-ACRIN Cancer Research Group. Protocol EA2234: A Randomized Phase II/III Trial of Intraperitoneal Paclitaxel Plus Systemic Treatment vs Systemic Treatment Alone in Gastric Carcinomatosis - STOPGAP II. clinicaltrials.gov; 2025. Accessed September 21, 2025. https://clinicaltrials.gov/study/NCT07001748

